# A Framework for Integrating Environmental Justice in Regulatory Analysis

**DOI:** 10.3390/ijerph8062366

**Published:** 2011-06-22

**Authors:** Onyemaechi C. Nweke

**Affiliations:** Office of Environmental Justice, U.S. Environmental Protection Agency, MC 2201A, 1200 Pennsylvania Avenue NW, Washington, DC 20460, USA;

**Keywords:** regulatory impact analysis, environmental justice, inequality, equity, regulatory development, inequity, disproportionate impacts

## Abstract

With increased interest in integrating environmental justice into the process for developing environmental regulations in the United States, analysts and decision makers are confronted with the question of what methods and data can be used to assess disproportionate environmental health impacts. However, as a first step to identifying data and methods, it is important that analysts understand what information on equity impacts is needed for decision making. Such knowledge originates from clearly stated equity objectives and the reflection of those objectives throughout the analytical activities that characterize Regulatory Impact Analysis (RIA), a process that is traditionally used to inform decision making. The framework proposed in this paper advocates structuring analyses to explicitly provide pre-defined output on equity impacts. Specifically, the proposed framework emphasizes: (a) defining equity objectives for the proposed regulatory action at the onset of the regulatory process, (b) identifying specific and related sub-objectives for key analytical steps in the RIA process, and (c) developing explicit analytical/research questions to assure that stated sub-objectives and objectives are met. In proposing this framework, it is envisioned that information on equity impacts informs decision-making in regulatory development, and that this is achieved through a systematic and consistent approach that assures linkages between stated equity objectives, regulatory analyses, selection of policy options, and the design of compliance and enforcement activities.

## 1. Introduction

Integrating environmental justice (EJ) considerations in all decisions is a priority for the US Environmental Protection Agency (EPA) [1,2]. In this regard, EPA recently developed a guide (“Process Guide”) that provides a legal and policy framework for considering EJ in its regulatory development process [3]. In this guide, EPA points to the development of technical guidance to “provide analytical tools and methodologies” for evaluating the impacts of EPA’s actions on minority, low income and indigenous populations as the next critical step. To advance to this next step requires foreknowledge and clarity about the equity objectives and questions driving each analysis, including how data so derived may inform the pending regulatory decision, all of which can in turn aid decisions about relevant tools and methods to assess equity impacts. Thus, for each proposed regulatory action, a clear description of the equity objectives is a requisite first step. Subsequently, it is pertinent that defined objectives are reflected in the tangible analytical activities that produce data used to inform regulatory design and options. This paper proposes a framework for regulatory analysis that enables this systematic thinking via emphasis on the development of: (a) a deliberate system of articulated and interrelated equity sub-objectives for each tangible step/activity in the regulatory development process, all linked to an overarching equity objective, and (b) activity-specific analytical/research questions written to ensure that stated equity sub-objectives and the overarching equity objective are met. A systematic process is important because it guarantees: consistent consideration of equity in the substantive steps of regulatory development; early and adequate planning for data collection and analysis; more focused assessments of equity impacts, deliberation about how equity can inform the specific regulatory decision at hand; and the conduct of equity assessments that meet clear decision making needs, all in a manner that is consistent across all relevant regulatory actions and transparent to all stakeholders.

Besides the recently developed “Process Guide”, there is no other known guidance to EPA analysts and decision makers on how to integrate equity in regulatory development. This lack of guidance is recognized in reports preceding the development of the “Process Guide” [4,5]. Although the “Process Guide” is seminal in that it offers a broad policy and legal framework for integrating EJ in the regulatory development process as a whole, additional direction can further focus analysts on the issues of structuring and relating analytical and decision steps in the process to yield necessary data on equity impacts. This framework aims to guide the analyst to develop a clear and consistent path throughout the process that results in a more focused use of limited resources for regulatory analysis, adequate planning for equity assessments, the conduct of equity assessments that are relevant to the pending decision, and proper utilization of resulting data in decision making.

The framework described herein punctuates key regulatory analysis steps with actions to drive explicit integration of equity, without suggesting a finite list of specific equity sub-objectives, analytical methods and policy considerations. This is because EPA’s regulations are established under a variety of statutorily driven legal and policy frameworks that generally prescribe or suggest the types of information (e.g., costs, technological feasibility, health risk, ecological risk, *etc*.) that are required to inform regulatory decisions. Developing a sufficiently exhaustive list of equity objectives, sub-objectives, and derivative analytical questions would at a minimum require in-depth analysis of each policy and legal context as well as analytical frameworks such as risk assessment, ecological risk assessment, and cost benefits analysis. Such analysis is quite extensive and beyond the scope of this paper. The expectation for the proposed framework is that irrespective of the operational legal and policy context or analytical framework, it prompts the analyst to generate pertinent objectives, sub-objectives, analytical questions and data.

Integrating EJ in regulatory development is an important step forward towards implementing the executive order on EJ (EO 12898). Regulations are the first line of action employed by the EPA to protect human health and the environment and have proved to be effective at preventing or mitigating exposure to harmful substances. In the United States for example, the Clean Air Act of 1970 required a phase-out of leaded gasoline by the mid-1980s. Between 1980 and 2008, resultant regulatory action reduced the national annual maximum quarterly average ambient air concentrations of lead by 92 percent [6]. Declines in air concentrations of lead have been associated with a decline in lead exposure for the US population; analysis of the Second National Health and Nutrition Examination Survey (NHANES II) blood lead data indicated a 37 percent drop in average population blood lead levels from February 1976 through February 1980, and a statistically significant correlation between levels of lead in blood and gasoline over this time period [7].

Similarly, environmental regulations can and should be used to target inequities in environmental health (EH) outcomes. Recommendations in the final report of the World Health Organization’s Commission on the Social Determinants of Health support this perspective. The Commission’s recommendations advocate: economic and social policy responses to climate change and environmental degradation that take health equity into account, coherent consideration of equity across all policies, and the assessment of the impact of all policies and programs on health and health equity [8]. This view concurs with the mandate of Executive Order (EO) 12898 to EPA and other federal agencies to identify and address social group differences in the health and environmental impacts of their policies, programs and activities. Therefore, in a post-executive order system, preventing new and reducing existing inequalities in EH outcomes that relate to regulation-specific pollutants or issues across social groups are companion priorities to aggregate and/or net benefits in environmental regulation. Regulatory development represents the first opportunity in a complex regulatory process for action to tackle the issue of inequities in EH outcomes. It is one of many policy processes including permitting, compliance, and enforcement, through which inequities in EH outcomes can be assessed and addressed.

## 2. Regulatory Analysis as a Tool and Process for Evaluating the Equity Impacts of Regulation

Regulatory analysis or Regulatory Impact Analysis (RIA) is a process for prospectively (and sometimes retrospectively) assessing the impacts of regulations. Theoretically, the results of assessments within a RIA should inform the development of regulations and/or the selection of a desirable policy option. The Organization for Economic Cooperation and Development (OECD) describes a RIA both as a policy tool and as a decision process for informing policy makers on whether and how to regulate to achieve public policy goals [9]. As a “tool” to support decision making, a RIA systematically examines potential impacts of government actions by asking questions about the costs and benefits; the effectiveness of the action at achieving its policy goals; and whether there are superior alternative options. As a “decision process”, RIA is integrated with systems of consultation, policy development and rule making within government in order to communicate information beforehand about the expected effect of regulatory proposals at a time and in a form that can be used by decision makers [9]. RIAs contribute to the policy-making process by promoting efficient regulatory policy and improved social welfare [10]. In the United States, the White House Office of Management and Budget (OMB) describes regulatory analysis as a tool regulatory agencies use to *“anticipate and evaluate the likely consequences of rules”* and further suggests that RIAs provide a formal way of organizing the key effects, both good and bad, of the various alternatives that should be considered in developing regulations [11].

The use of RIA to inform environmental policy-making is a standard practice in the US. It is required for certain types of regulations under the “Regulatory Right-to-Know Act” of 2000 and Executive Order 12866 of 1993 entitled “Regulatory Planning and Review”. With respect to equity, EO 12866 specifies that *“…in choosing among alternative regulatory approaches, agencies should select those approaches that maximize net benefits (including potential economic, environmental, public health and safety, and other advantages; distributive impacts; and equity), unless a statute requires another regulatory approach.”* Thus, EO 12866 embraces social welfare considerations [12] and advocates the outcome of equity as one of different types of benefits that can inform regulatory approaches to an issue. Furthermore, interpretive guidance for EO 12866 (Circular-A4) notes that regulations may have distributive effects across the population and economy, and recommend that *“regulatory analysis should provide a separate description of distributional effects (i.e., how both benefits and costs are distributed among sub-populations of particular concern) so that decision makers can properly consider them along with the effects of economic efficiency.”* [11] Executive Order 13563 of 2011 reaffirms EO 12866, and specifically encourages federal agencies to consider equity, fairness and distributive impacts.

In practice, the RIA process may be conflated with specific analytical tools, and particularly cost benefit analysis (CBA) [13]. The presumption in the framework proposed herein is that CBA provides one set of analytical outputs that may or may not be required for decision making given statutory requirements. Therefore, this framework offers a system for consistently evaluating equity impacts in the context of different analytical frameworks (e.g., risk assessment, ecological risk assessment) that are used to inform decision making in the context of regulatory development. This framework also ensures proactive consideration of equity in planning, scoping and executing analyses within an RIA, and also that equity considerations consistently inform the selection of one policy option/regulatory approach over others.

The formal process for developing most regulations in the United States is known as the Action Development Process (ADP). The ADP is designed to ensure that all statutory and administrative requirements for rulemaking are met [14]. The RIA process at EPA is embedded in the ADP. Details about EPA’s ADP process can be found in its recent guidance *“EPA’s Action Development Process: Interim Guidance on Considering Environmental Justice During the Development of an Action”* [3].

## 3. A Framework for Incorporating Equity Considerations in Regulatory Impact Analysis

### 3.1 The OECD Regulatory Impact Analysis Framework

**Figure 1.**
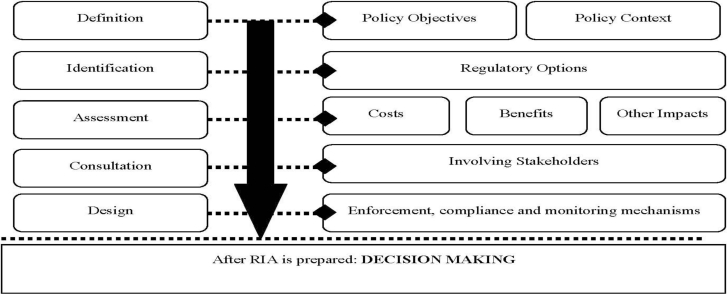
OECD Framework for Regulatory Impact Analysis. Source: Building an Institutional Framework for Regulatory Impact Analysis (RIA): Guidance for Policy Makers [10].

The proposed framework for integrating equity in the RIA process builds off the structures and elements for an RIA process and impact assessments that are outlined in the recent guidance documents published by the OECD and the European Commission respectively [10,15]. The OECD framework articulates five sequential stages in an RIA (Figure 1): defining the policy context and objectives; identifying regulatory options; conducting assessments that are required to inform the process of selecting a policy option; consultation with stakeholders; and design of enforcement, compliance, and monitoring mechanisms [10]. The proposed framework is based on the OECD framework because the latter focuses on key activities in regulatory development, activities that are substantively relevant in terms of informing the analytical and decision making process. As noted earlier, these key activities are embedded in the various stages of EPA’s ADP. For example, EPA’s ADP process includes a planning process during which major issues that need to be addressed by the regulation are identified. It also includes a stage for identification of data needs and plans to collect data, analytical steps, and also a phase for evaluating regulatory options [3].

**Figure 2.**
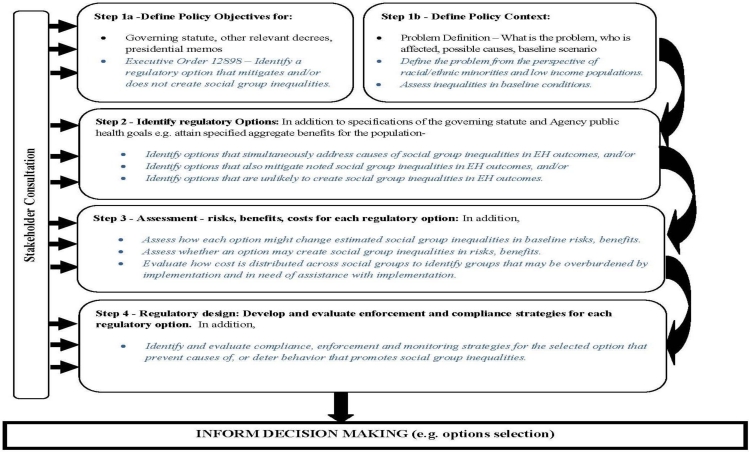
Proposed Framework for Assuring the Integration of EJ in Regulatory Impact Analysis.

**Figure 3.**
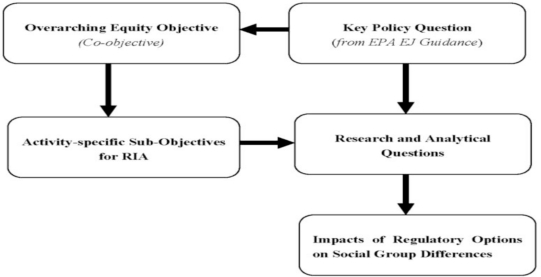
Relationship between Overarching Objectives, Sub-objectives and Research and Analytical Questions in Proposed Framework.

The proposed framework retains the five element structure of the OECD framework, the primary difference being that stakeholder input feeds into all stages of an RIA (Figure 2) in concordance with EPA’s policies on public participation and meaningful involvement. Within the framework, I propose overarching equity objectives and subsequently highlight derivative sub-objectives in each element of the RIA process that are designed to focus attention on equity at each stage. These sub-objectives are included in the framework to assure that the appropriate questions about equity are asked of the analysis. The logic of the framework in terms of the relationship between objectives, activity-specific sub-objectives in the RIA, and research and analytical questions is shown in Figure 3. The intended message in proposing the framework is that policy objectives ought to be linked to the problem description, the analysis, selection of policy options, comparison of policy options and planned compliance and evaluation activities [15]. The relevance of properly framing the policy and analytical questions cannot be overstated; an RIA’s usefulness depends “mightily” on how the questions are structured [12].

The presentation of the proposed framework does not include a discussion of the role of stakeholder consultation. The rationale for this is two-fold. First, the paper maintains a weighty focus on advancing the integration of EJ in the analytical steps of the framework. Second, there is ample direction in the recently developed guidance by EPA [3] on engaging stakeholders, and in particular affected communities, throughout the process of regulatory development, which includes steps in an RIA. In addition, EPA has other extensive resources and tools on stakeholder involvement [16–18] that are directly relevant to stakeholder participation in the RIA process. Furthermore, the agency has established public participation requirements for specific regulatory development activities as highlighted in a 2000 review of its public participation policy and regulations [19]. The presumption in this paper is that community knowledge and expertise on a subject can significantly improve regulatory outcomes, especially because of stakeholders’ local scale appreciation of environmental problems for which regulation is proposed, and that these existing processes and resources will be applied to guarantee meaningful stakeholder contributions to the RIA process.

The proposed framework may be unfamiliar to analysts who do not readily identify with the abbreviated arrangement or adopted terminology. However, the logic of the framework is fundamentally unchanged relative to EPA’s regulatory development process with respect to the order of these key activities. Therefore, to utilize the proposed framework, analysts and policy makers should aim to identify with relevant activities within the framework.

To refer to disproportionate EH outcomes in the suggested objectives, sub-objectives, and analytical/research questions the term “inequalities” is used. This merits some explanation given that differences in the context of EJ are considered unfair and unjust, and therefore inequitable. The concept of fairness and justice that is implied in the definition of EJ has many definitional variants that contribute to the perspective that EJ as a concept is vague, abstract and difficult to define in practical real-world terms with implications for quantitative analysis [20]. By using the term “inequality”, an analyst is unlikely to be overwhelmed with dissecting the fairness and justice elements of a measured difference.

The definitions of inequalities and inequities have evolved such that inequalities refer to any quantitative differences between groups or individuals whereas inequities are differences that are unnecessary and avoidable, unfair and unjust [21], and systematic and potentially remediable [22]. Equity entails normative judgment based on the analyst’s theories of justice and society, and their reasoning about the underlying genesis of inequalities, whereas inequality is a descriptive term that need not imply moral judgment; therefore concerns about equity dictate that inequalities across social groups are evaluated and with due consideration of normative judgments [23]. In EJ, social group differences of interest are presumed to have evolved from unfair or unjust circumstances. Therefore, in measuring these social group inequalities in EH outcomes in the context of environmental regulation, one could argue that the differences measured are mostly those considered unfair and unjust. Levy *et al*. [24] note that socially constructed elements of fairness and justice transcend the scope of mathematical analysis. They surmise that equity in the context of EJ does not lend itself to quantification and advocate that ultimately inequalities in EH outcomes are what concern EJ advocates.

Indeed inequalities in EH outcomes are the bottom-line yardstick for measuring disproportionate adverse environmental conditions in overburdened communities or populations in the literature [25–30], and is the terminology adopted in this paper. However, the use of the term inequalities should not preclude consideration of value judgments that pertain to inequities which may be important in decisions about methods for quantifying these differences.

#### Step 1a—Define the Policy Objectives

Objectives drive the approach adopted for an RIA process, and are critical to determining data needs and types of research and analytical questions raised. Without a clear understanding of what a future policy is supposed to achieve, it is difficult to identify courses of action, and even more difficult to compare policy options. Objectives provide the only effective criteria for assessing the success or failure of the proposed policy options [15].

The primary basis for environmental regulation in the US derives from the environmental statutes such as the Clean Air Act (CAA). These types of statutes are herein referred to as “governing statutes”. Environmental regulations are also subject to other statutes (e.g., the Regulatory Right-to-Know Act) or presidential decrees (e.g., EO 12898), herein referred to as “secondary statutes/decrees”. In general, environmental statutes and decrees can be prescriptive, providing EPA with directives on a mechanism to solve a problem (e.g., set a standard), and how to proceed with putting the mechanism in place. Therefore, it is typical for the main objectives of environmental regulation to be provided by the statutory authority. For example, Section 109 (42 USC 7409) of the CAA requires the EPA Administrator to set National primary Ambient Air Quality Standards (NAAQS) that allow an “*adequate margin of safety*”, and “*are requisite to protect the public health”.* Through the language in the CAA, EPA is provided with a key objective/goal, which is to set a NAAQS that allows an adequate margin of safety, and that is requisite to protect public health. Because environmental regulations are also subject to secondary statutes/decrees, it is necessary to identify policy “co-objectives” that evolve from them. EO 12898 is one of such secondary decrees.

The European Commission Impact Assessment guidelines lay out helpful principles for setting good objectives: objectives should be clearly linked to the problem; general objectives should be identified and translated into specific and as appropriate, operational objectives; and objectives should be measurable, achievable, realistic and time-dependent [15]. The equity objectives for any regulatory action undertaken by EPA are embodied in the three questions developed by the EPA to assure the consideration of EJ [3]. In particular, the second question *“How do you plan to (or how did you) address existing and new disproportionate environmental public health impacts on minority, low income and indigenous populations during the rule making process?”* is insightful about how the analytical co-objectives and related activity-specific sub-objectives can be defined. The term “disproportionate” sets the expectation that a comparison to some reference group is necessary, and that the adverse effects in question should be measurably different between the social groups that are the focus of the executive order and identified reference group(s). Given the language of the executive order, obvious reference groups are non-minority or higher income groups, although comparisons using other socio-demographic classification schemes are plausible depending on the objectives and analytical questions. I refer to social group inequalities throughout this framework in line with the language of the executive order given that fair treatment is a key concept in EJ. Therefore, conceptually a meaningful disproportionate difference between social groups is actionable independent of the group bearing the disproportionate burden. Finally, the reference to “new” and “existing” EH outcomes highlights current and future inequalities as important considerations.

Two possible examples of policy co-objectives evolve from the recent EPA guidance and the language of the EO and are used to illustrate the proposed framework: (1) identify a regulatory option that does not create inequalities in EH outcomes (e.g., human exposure and risk) across social groups associated with the regulated source/pollutant; and (2) identify a regulatory option that mitigates existing inequalities in EH outcomes (e.g., human exposure and risk) across social groups associated with the regulated source/pollutant. The first co-objective addresses the issue of prospective inequalities in the impacts of proposed regulation. The second co-objective focuses on existing inequalities. These co-objectives relate to the key policy question discussed above that was developed by EPA to assure the consideration of EJ in regulatory development. Although the stated co-objectives are written to address exposure and risk, they are easily adaptable within the context of other types of impacts.

#### Step 1b—Define the Policy Context

Providing the context for proposed policy requires a definition of the problem for which regulatory action is planned [10]. Problem definition is the most important step in public health problem solving, and has consequences for problem measurement, policy making and evaluation [31]. The process of defining a problem entails several activities that include: a review of laws and statutes to define the legal framework for the regulatory action; identifying causes of the problem (drivers/determinants/root causes of the problem); description of the nature and scale of the problem (what is the extent of the problem for which regulation is proposed, which population groups are most affected, and what are the risks of specific adverse health events associated with different levels of the exposure); and assessment of the baseline scenario (e.g., what conditions look like without regulatory action) [15,32]. These types of activities occur in different constructs and to different degrees depending on the regulatory structure. For example, the NAAQS process mandates the development of the Integrated Science Assessments, which include thorough and extensive statutorily mandated problem definition exercises, particularly in terms of describing affected groups, associated health risks and vulnerable populations. For the activities that characterize the task of defining the policy context, a variety of equity sub-objectives and related research and/or analytical questions are possible (Table 1). One example of a reasonable sub-objective for activities that characterize this phase in the RIA could aim to identify baseline racial/ethnic and income inequalities in EH outcomes such as risk and/or exposure, which in turn can drive the consideration of cumulative burdens and vulnerability typically experienced by racial/ethnic minority and low income groups in the regulatory development process for health protective regulations [24]. A related research question might direct the analyst to identify social group differences in baseline prevalence rates for diseases that are associated with the exposure(s) of interest, and/or estimate social group differences in baseline risks directly attributable to the exposure in question. Data from the analysis can subsequently feed into other analyses and decisions in the RIA to meet the overarching co-objectives for the regulation. Other types of questions could direct the analyst to identify determinants of inequalities that are subsequently helpful for thinking through regulatory options and even compliance mechanisms. For example, environmental racism and income are two drivers of inequality [27,28,33] likely to be identified by EJ stakeholders. However, the direct application of such information in the context of regulatory development may not be feasible because of the structure of environmental statutes. Nevertheless, regulations are implemented by states, localities and other institutions, and via multiple policy actions. More importantly, policies, institutions and private decision-making are increasingly becoming interactive, and decisions of private individuals are often responsive to institutional arrangements and public policies in ways that perpetuate discrimination [34]. Therefore, racially- or income-driven inequalities in EH outcomes may be unintended and undesirable outcomes of regulatory policy. Understanding these complex interactions is valuable because such knowledge can unveil aspects of the regulatory process that are vulnerable to such influences, or that facilitate the perpetuation of the historical influences of these drivers; knowledge of weaknesses in the regulatory approach can inform the design of regulations as well as implementation, compliance and enforcement.

#### Step 2: Identify Regulatory Options

A regulatory option is the proposed solution to an identified problem. The process of identifying solutions may require evaluating environmental technologies, changes in environmental management practices, and incentives that can motivate better environmental performance [14]. Policy options should be proportionate to the extent of the problem, include the option of not to regulate, and should be clearly linked to the causes of the problem and the objectives of the regulatory exercise [15]. The examples of sub-objectives and research questions provided in Table 1 illustrate how equity may be integrated into this step of a RIA.

The sample sub-objectives force explicit consideration of options that meet primary goals of regulation, and also potentially impact social group differences in EH outcomes or do not result in the same. With the first sample research question, the analyst and decision maker base the selection of a range of proposed ambient media concentrations *i.e.*, the regulatory options, on the potential for such options to impact both equity and other goals of the regulation. The second sample question focuses on mechanisms through which an option addresses inequalities in order to help the analyst and decision maker create/identify options that target pre-identified determinants of EH inequalities such as management practices. These examples also highlight the comprehensive integration of equity during problem formulation (step 1b) as an important exercise in order to access the type of information needed to assure that equity considerations influence identification of options.

#### Step 3: Assessing Risks, Benefits, *etc.* for Each Regulatory Option

The assessment phase of a RIA is the most data-demanding aspect of the process. It depends on preceding steps in the process, and generates most of the information needed by the decision makers to compare identified options and inform the selection of a preferred option. Impact assessments are used to highlight a variety of direct and indirect as well as intended and unintended types of impacts (economic, equity, *etc*.), how they occur and who is affected [15]. In a typical RIA, a comparison to evaluate policy options takes the form of a pre- and post-policy assessment of risks, benefits, and other impacts. When social group inequalities are under consideration, the pre- and post- assessment should also focus on how health risk inequalities change with and without each proposed regulatory option. To encourage analysts to consider these types of comparisons, the analyst could introduce the sample sub-objective that is specific to the issue of how each option is predicted to change identified baseline social group inequalities. Related analytical questions provide insight into the types of information that are required to decide on the potential for any specific option to adequately address social group inequalities. Specifically, these questions can be used to focus attention on the specific issues of changes in baseline social group inequalities in EH outcomes, the magnitude of any observed change, whether the change is meaningful and therefore actionable (*i.e.*, the basis to select one option over the other), and the direction of the change to address concerns about risk transfers. Besides assuring equity comparisons of policy options, these types of questions also influence methodological issues such as which inequality measure is most appropriate for the analysis and what reference group is appropriate for the analysis. This is an important discussion that is beyond the scope of this paper; nevertheless insights into these issues are available in the inequality literature [24,35].

#### Step 4: Regulatory Design—Compliance and Enforcement

Regulations are only likely to attain the desired objectives if there is a high degree of compliance, and adequate enforcement when there is non-compliance. EPA assures compliance with US environmental regulations through mechanisms such as compliance assistance, compliance monitoring, and compliance incentives and auditing. EPA uses compliance assistance to help regulated entities comply with environmental laws and contends that it is most effective when used in an integrated strategy that combines compliance monitoring (inspections), compliance incentives and auditing (self-disclosure policies), and enforcement [36]. Sound regulatory enforcement promotes welfare in a variety of ways such as protecting environmental media [37].

The examples of sub-objectives and analytical questions for this step illustrate how analysts might evaluate how compliance and enforcement activities mitigate or prevent inequalities. In the design of compliance and enforcement mechanisms, assurance that the co-objective of mitigating or preventing inequalities is considered can be achieved through questions about costs of implementation. For example, there could be scenarios in which a regulation requires changes to a specific service that is offered for a fee to consumers, and the costs of implementing the new regulation are subsequently transferred to the end user. Affordability issues with the higher costs may cause some consumers to opt out from receiving the service, and therefore preclude them from receiving the benefits of the regulatory action. How this continues existing inequalities in related EH and other outcomes, or creates new ones is information that is useful in the decision making process, and is captured in some of the sample questions for this step. Another example of a regulatory design issue with respect to inequalities that can be addressed through a sub-objective is identifying compliance approaches that best deter corporate behavior patterns that are identified in the problem definition stage as promoting inequalities, and also that prevent conditions that promote such negative behavior. In this example, the design of regulation is linked to information gathered in the earlier stages of the RIA process on determinants of inequalities. Key determinants identified during the RIA process (e.g., during problem definition) can inform the design of more effective compliance requirements and enforcement strategies. Such knowledge may also highlight opportunities for other types of mitigation strategies (including non-regulatory options) to address inequalities.

## 4. Discussion and Conclusions

The successful integration of EJ in environmental regulatory decision-making at EPA depends on the convergence of several lines of instruction including guidance on: framing regulatory analysis to assure adequate integration of EJ and the usefulness of resulting information, methods and data selection, and policy issues that underlie choice of methods and inform decision making. The proposed framework is designed to be instructive about the specific issue of framing regulatory analysis. It is purposed to help analysts and decision makers conduct equity evaluations that are connected to the decisions they have to make in the regulatory development process. The proposed framework emphasizes the following key actions: (a) defining an equity objective for the proposed regulatory action at the onset of the regulatory development process; (b) identifying activity-specific objectives when planning key analytical and decision steps/activities in the RIA process, to assure that the intent in the overarching equity objective is captured throughout the entire process; and (c) developing explicit analytical/research questions around each activity-specific objective to assure that these goals are met. This logic is further illustrated through examples of equity objectives, sub-objectives for key activities in the RIA process, and analytical/research questions specific to each sub-objective, and using an adaptation of the OECD RIA framework. These examples are generic and not exhaustive given that they were not developed under any regulatory context, and are therefore solely illustrative. Nonetheless, they capture concepts that are pertinent to equity analysis in many contexts in regulatory development. Accordingly, they can be viewed as “building block” questions to be adapted to reflect the context of each regulatory action that is the focus of a regulatory assessment.

In structuring a RIA to integrate equity concepts, analysts and decision makers are bound to confront policy questions/issues that directly influence the analytical process or even the outcome of the RIA. Although these policy issues cannot be adequately reviewed given the scope of this paper, they merit some mention. One of such questions is how to address disproportionate impacts that accrue to non-minority or higher income groups. Indeed, while minority and low income groups tend to generally bear disproportionate environmental health impacts, the reverse is plausible in reality. As indicated earlier, environmental justice embodies the concept of fair treatment, which means that no group of people should bear a disproportionate burden of environmental harms and risks [3]. By inference, inequalities that are sufficient to trigger a policy action are actionable irrespective of who bears the disproportionate burden. Also, the likelihood that non-minority or higher income groups may bear a disproportionate environmental health burden advocates for structuring equity assessments that are not blind to this information. For example, some analysis may be framed to compare the proportion of minorities resident around a certain type of polluting entity to the proportion of minorities nationally. This type of limited comparison potentially blinds the analyst to high burdens borne by non-minority groups. While such a comparison is not in error, it certainly would benefit from an additional direct comparison between social groups. Such oversight can be avoided by systematically structuring analysis.

A second policy issue for decision makers and analysts to grapple with is whether inequalities that occur below an acceptable risk threshold are actionable. A determination could be made that the regulatory options under consideration will reduce risk for the most exposed to a level that is below an acceptable risk threshold (e.g., cancer risk of 1 in a million). In that instance, should a decision maker proceed with an equity assessment? Also, an early determination could be made based on available technology options that the least stringent regulatory option will reduce risk for the most exposed to a level below an acceptable threshold. Should the decision maker still evaluate which of the regulatory options has the most impact on social group inequalities? What if there is a small cost differential between the least and most stringent regulatory options and the most stringent option has the most impact on social group inequalities, should a decision maker act on such information? A decision not to conduct an equity assessment of regulatory options can be driven by knowledge that the estimated risks fall below an “acceptable risk” or “presumed safe” threshold. A possible conclusion is that since estimated post-regulatory option risks for all in the population are predicted below a threshold of concern, there is no outstanding “adverse effect”, and therefore no need for further evaluation of social group inequalities. However, from a public health perspective, this scenario merits further consideration in terms of the policy implications. One perspective to consider is that if an exposure cannot be determined with certainty to be associated with zero risk, prudence will dictate that the overall goal should be to reduce exposure for all in the population, and especially the most exposed or most vulnerable, to the extent that is feasible and practical. Thus, a potential policy position that evolves from such reasoning is that under conditions where all risks are below an acceptable threshold, significant social group inequalities cannot be considered meaningless and should not be ignored. Finally, the appropriate policy action to adopt when inequalities in exposures and risks occur below established policy thresholds further raises other important science and policy questions about the extent to which underlying data for estimating exposure-response relationships account for issues like the cumulative contributions of multiple stressors to risk potential across different population groups, and the extent to which these vulnerabilities are adequately accommodated by these thresholds. These types of concepts can only be explicitly evaluated if the appropriate questions about equity are integrated in the relevant steps of the RIA, hence the need for a systematic approach to think through the steps of an RIA from an equity perspective.

The issue of cumulative risk and how to account for it in regulatory development is yet another important policy issue in the context of integrating equity in the RIA process. There is evidence to suggest that social stressors interact with chemical hazards to yield higher risks of adverse health outcomes [38–40]. These data affirm the concerns of EJ stakeholders who have always advocated the use of cumulative risk data in decision making [41]. Information on cumulative risk/interactive effects offers a range of insights about potential risk inequalities and opportunities to address them that may otherwise not be evident. For example, quantifying cumulative risk may unveil social group differences in cumulative cancer risks to all sources where there are no inequalities in cancer risk from a single regulated chemical stressor or source. Also, evidence of risk modification by a second factor or condition may prompt the identification of regulatory options that can directly or indirectly address more than one stressor or source. However, the science of cumulative risk estimation and interactive effects and the methods for applying such information in decision making are in need of further advancement, although the cumulative risk assessment approach can be qualitative. Nevertheless, given the usefulness of these concepts for evaluating and addressing equity concerns, it is an area worth embracing in decision making albeit with careful deliberation about technical feasibility (e.g., data, methodology) and applicability of resulting data within any given operational legal and policy framework. Cumulative risk is certainly a key issue to EJ stakeholders, and has the potential to better inform decision making related to pollution management and risk reduction. Although cumulative risk assessment and its integration in regulatory development may seem daunting, there is at least one proposal for an approach to advance its application in risk-based decision making that should be considered [41].

The appropriate range of adverse health endpoints that should be assessed in an EJ evaluation of a regulatory action can have great implications for the scale of an assessment. Potential key questions for the analyst and decision maker in this regard could be of the nature “Should an assessment focus on all adverse health impacts for which data is available?”, or “should an analysis limit the consideration of adverse health outcomes to those resulting from the specific pollutant(s) in question?” In some instances it may prove valuable to understand how any one regulatory action may change overall population risk profile or disease burden and any social group inequalities in those outcomes. Perhaps such analysis can provide insights about the extent to which environmental regulations and programs improve overall health status and mitigate inequities, and therefore highlight opportunities for other supplemental non-regulatory actions. From a pragmatic point of view however, the most relevant question in the context of the decision to be made in a regulatory development process relates to the impacts of a regulatory action on outcomes that are associated with the regulated pollutant or entity. Importantly, focusing analysis on the spectrum of effects associated with the source/pollutant does not negate the use of information on other burdens or risks experienced by the target population (such as would be provided in a cumulative risk assessment) to inform decision making. Defining the range of adverse effects for an equity impact assessment within a RIA is worth early contemplation in the process; in fact, it is possibly an issue for which a blanket policy for all regulatory development activities is valuable.

Lastly, conflicting policy goals in the context of regulatory development can emerge in the course of conducting a RIA, such as when results of an economic analysis may suggest that attaining equity conflicts with other desirable policy goals. Using an earlier example in the section on regulatory design, attaining equity may demand more costly choices that are passed through to the beneficiary of the regulatory action. For populations already resource-strapped, this might present the option of opting out of benefiting from the regulatory action, or where they are not permitted the choice to opt out, limited access to another necessity (*i.e.*, the opportunity cost principle). That these scenarios are likely supports the need for proactive and systematic consideration of the impacts of a regulatory action during the process of analyzing regulatory impacts and designing regulations. Such deliberate evaluation may for example inform the adoption of non-regulatory approaches to support the implementation of the regulatory action, an unlikely consideration if equity issues are not systematically and overtly evaluated throughout the RIA process.

The policy issues discussed thus far reflect the complexity of equity considerations and have far reaching implications for how equity is integrated in regulatory analysis. It is important to note that they represent a sampling of the types of critical science-policy questions that would have to be addressed to successfully integrate EJ in regulatory development. In addition to policy issues are several technical issues, some of which are alluded to in this paper. Examples include: identifying methods for quantifying inequalities that embody EPA’s normative and policy decisions; addressing issues of equity weighting in economic analysis and its applicability in assessing EJ impacts, which also will reflect ethical and policy preferences; and issues related to the adequacy of available data on willingness-to-pay to avoid risk or benefit from regulation in the context of assessing for EJ impacts in regulatory development. Efforts to tackle these policy, science and methodological issues can benefit from previous experiences within the United States and globally with integrating equity in policy making. Specifically, existing guidance and framework documents such as EPA’s 2000 draft guidance for conducting investigations of Title VI administrative complaints challenging permits [42], Council on Environmental Quality’s (CEQ) guidance on EJ under the National Environmental Policy Act [43], the recently developed Equity Focused Health Impact Assessment framework [44], and other directions and resources on health impact assessment [45,46], as well as analytical approaches demonstrated by researchers [24,47–54]are resources that can provide helpful perspectives.

As equity becomes a mainstream concept in environmental regulation, consideration of a broader range of inputs in regulatory assessment from various disciplines and lay experts, and paradigm shifts in both technical and policy approaches to regulatory development can be expected. For this reason, efforts to advance the consideration of EJ in regulatory development is necessarily multidisciplinary and must seek the input of multiple stakeholders, and especially EJ stakeholders whose local knowledge and expertise on issues such as exposures, health effects, compliance behavior, local governance issues, *etc*. as well as value systems and preferences are likely to prove insightful. Successfully harnessing these types of information from stakeholders calls for a structured plan for meaningful engagement at all phases of the RIA process, and will benefit from full implementation of EPA’s public participation policies and frameworks.

In concluding, it is necessary to recognize that regulatory development process is characteristically complex which begs the question, “Why additional complexity and detail?” The extra level of detail advocated in the proposed framework is important given the novelty of and unfamiliarity with the concept of equity in regulatory development, and would help build confidence in policy recommendations that are partly based on equity impacts. The next step toward implementing this framework is to align it with analytical frameworks (e.g., risk assessment, ecological assessment, *etc*.) and to develop sub-objectives and analytical questions that are specific to each analytical framework. The potential architecture of these questions is currently reflected in some of the examples used in the proposed framework. By creating a reservoir of analytical questions, analysts need only grapple with framing these questions within their respective regulatory contexts and assuring that the questions address their pre-defined sub-objectives.

## Figures and Tables

**Table 1.** Examples of Sub-objectives and Correspondent Analytical/Research Questions for Key Steps in Regulatory Impact Analysis.

**Activity**	**Equity Sub-Objective**	**Equity-focused Analytical/Research Question(s)**
**Define the problem—identify determinants, describe scope and scale of problem, identify populations most affected, estimate baseline scenario**	Identify inequalities in EH outcomes (e.g., exposure to source and/or hazard and related risks) from all sources. Identify inequalities in EH outcomes related to the regulated source or hazard, or regulatory action. Identify determinants and causal factors for inequalities in EH outcomes that can be remedied via the proposed regulatory action.	Do minority and low income populations experience higher risks of disease conditions associated with exposure(s) in question? Are minority and low income populations more proximate to regulated sources in question? How are EH outcomes distributed in the baseline scenario across social groups? What is the nature and magnitude of observed racial/ethnic and income inequalities in the baseline distribution of EH outcomes? What are identified risk factors /determinants of EH outcomes in the literature? Which determinants are directly or indirectly remediable within the regulatory structure for the proposed regulation?
**Identify regulatory options**	Identify options that yield maximum benefit in terms of aggregate improvements in social welfare and decrements in racial/ethnic and income inequalities. Identify options that do not cause social group inequalities in EH outcomes.	What level of reduction in pollutant concentrations in ambient media reduce social group inequalities and also yield desirable benefits for the population? What are the proposed mechanisms through which an option address one or more causes or determinants of social group inequalities in EH outcomes?
**Assessment—risks, benefits, costs related to identified options**	Describe how each option is predicted to change identified baseline social group inequalities in EH outcomes.	What is the change in estimated baseline racial/ethnic and income inequalities in EH outcomes (e.g., risk) given implementation of a specific option? Given a decrease in inequality in risk from baseline conditions, what is the magnitude of the observed decrease? Is the decrease meaningful? How does the decrease occur? How are health benefits distributed across social groups?
**Develop and evaluate compliance, monitoring and enforcement strategies**	Describe how the costs of implementing each option are distributed across income groups. Identify compliance, enforcement and monitoring strategies for the selected option that deter behavior or activities that promote social group inequalities.	How are implementation costs distributed across income groups? Are compliance costs across distributed unequally across income groups Are the inequalities significant and a potential hindrance to attaining desired objectives of regulation (e.g., health protection, reduction in inequality in EH outcomes)? Do compliance strategies target applicable identified causes/determinants of social group inequalities? Can compliance strategies deter identified behavior of the regulated entity that promotes social group inequalities?
